# Evaluating breast ultrasound S-detect image analysis for small focal breast lesions

**DOI:** 10.3389/fonc.2022.1030624

**Published:** 2022-12-13

**Authors:** Boyuan Xing, Xiangyi Chen, Yalin Wang, Shuang Li, Ying-Kui Liang, Dawei Wang

**Affiliations:** ^1^ Department of Ultrasound Imaging, The People’s Hospital of China Three Gorges University/the First People’s Hospital of Yichang, Yichang, Hubei, China; ^2^ Department of Nuclear Medicine, First Affiliated Hospital of Guangxi Medical University, Nanning, China; ^3^ Department of Medical Engineering, Medical Supplies Center of PLA General Hospital, Beijing, China; ^4^ Department of Pathology, The People’s Hospital of China Three Gorges University/the First People’s Hospital of Yichang, Yichang, Hubei, China; ^5^ Department of Nuclear Medicine, The Sixth Medical Center of People's Liberation Army General Hospital, Beijing, China

**Keywords:** S-detect, breast imaging reporting and data system, BI-RADS, breast cancer, ultrasound

## Abstract

**Background:**

S-Detect is a computer-assisted, artificial intelligence-based system of image analysis that has been integrated into the software of ultrasound (US) equipment and has the capacity to independently differentiate between benign and malignant focal breast lesions. Since the revision and upgrade in both the breast imaging-reporting and data system (BI-RADS) US lexicon and the S-Detect software in 2013, evidence that supports improved accuracy and specificity of radiologists’ assessment of breast lesions has accumulated. However, such assessment using S-Detect technology to distinguish malignant from breast lesions with a diameter no greater than 2 cm requires further investigation.

**Methods:**

The US images of focal breast lesions from 295 patients in our hospital from January 2019 to June 2022 were collected. The BI-RADS data were evaluated by the embedded program and as manually modified prior to the determination of a pathological diagnosis. The receiver operator characteristic (ROC) curves were constructed to compare the diagnostic accuracy between the assessments of the conventional US images, the S-Detect classification, and the combination of the two.

**Results:**

There were 326 lesions identified in 295 patients, of which pathological confirmation demonstrated that 239 were benign and 87 were malignant. The sensitivity, specificity, and accuracy of the conventional imaging group were 75.86%, 93.31%, and 88.65%. The sensitivity, specificity, and accuracy of the S-Detect classification group were 87.36%, 88.28%, and 88.04%, respectively. The assessment of the amended combination of S-Detect with US image analysis (Co-Detect group) was improved with a sensitivity, specificity, and accuracy of 90.80%, 94.56%, and 93.56%, respectively. The diagnostic accuracy of the conventional US group, the S-Detect group, and the Co-Detect group using area under curves was 0.85, 0.88 and 0.93, respectively. The Co-Detect group had a better diagnostic efficiency compared with the conventional US group (*Z* = 3.882, *p* = 0.0001) and the S-Detect group (*Z* = 3.861, *p* = 0.0001). There was no significant difference in distinguishing benign from malignant small breast lesions when comparing conventional US and S-Detect techniques.

**Conclusions:**

The addition of S-Detect technology to conventional US imaging provided a novel and feasible method to differentiate benign from malignant small breast nodules.

## Introduction

Breast cancer is the most diagnosed cancer and the leading cause of cancer mortality in women worldwide. Despite significant advances in screening, imaging, and surgical and adjunctive treatment, the management of breast cancer exacts a costly and psychological toll (1). Recently, breast cancer has become the most frequently diagnosed cancer in Chinese women (30.4 cases per 100,000; estimated incidence rate of 17.1%) (2). Ultrasound (US) examination is a valuable and commonly used technology for breast screening that has numerous advantages including relatively low cost, real-time imaging, no radiation exposure, millimeter-level resolution, and the opportunity for interventional applications. US evaluation is principally utilized to characterize two-dimensional lesion morphology, but can be extended to use elastography or acoustic radiation force impulse imaging (ARFI) to assess tissue stiffness, and three-dimensional US (3D-US) can yield more detailed imaging. As part of the breast cancer screening armamentarium, US has contributed to earlier diagnosis, which facilitates more conservative and more effective treatment with commensurate improved patient outcomes (2, 3). Tumor stage is a critical component of patient survival, as illustrated by the 10-year overall (OA) survival rate of 81%–93% in patients with breast cancers measuring ≤2 cm, compared to the OA rate of 60%–70% with cancers >2 cm (4, 5). Small breast nodules can have considerable variability in the shape, margin appearance, and echo pattern by conventional B-mode US that causes difficulty in distinguishing benign from malignant lesions (6, 7). Breast imaging-reporting and data system (BI-RADS) is the standard US lexicon aimed at standardizing image interpretation, reporting, and teaching breast imaging. S-Detect is a computer-assisted embedded diagnostic software program based on a deep-learning algorithm (without image postprocessing and therefore not influenced by human bias) that automatically analyzes the inner constructions, refracted echoes, interfaced regions, morphological manifestations, and other parameters of the specific breast nodule (8, 9). Hence, the intention of the present study was to investigate the statistical performance of conventional US, S-Detect technology, and the two techniques in combination (Co-Detect) in distinguishing benign from malignant small breast nodules (≤2 cm) as confirmed histologically.

## Materials and methods

### Study design

The study was carried out in accordance with The Code of Ethics of the World Medical Association (Declaration of Helsinki). Institutional review board approval and informed patient consent were obtained for this prospective study. Permission was obtained from the hospital for patient medical records review.

Between January 2019 and June 2022, 326 nodules (size range: 0.30–2.00 cm; mean, 1.19 cm ± 0.43 SD) were detected in 295 women (age range: 18–90 years; mean, 45.40 years ± 11.40 SD) from the Department of Ultrasound or the Department of Thyroid and Breast Surgery in the First People’s Hospital of Yichang city. Indicators for breast US and BI-RADS included the following: (1) a palpable lesion with a diameter ≤ 2 cm, (2) lesion tissue obtained by biopsy or conventional surgical excision, and (3) a definite pathological diagnosis. Exclusion criteria were as follows: (1) anamnesis and prior breast tissue biopsy or surgery, and (2) pregnant or breast-feeding patient.

### Instruments and strategies

The US machine used was a type RS80A (Samsung) equipped with an L2-12A ultrasonic probe (5–13 MHz). The patient was positioned supine, the appropriate breast was fully inspected, and multi-slice and stereo scans from each quadrant were obtained. An independent experienced US radiologist/operator conducted the sonography, measured the maximal diameter of the index lesion(s), and assessed the adjacent tissue relationship of each lesion. Records of the size, morphology, orientation, inner-echo density, calcification(s), blood flow, homolateral lymph node metastases, and key sonographic features were kept. Then, US scan mode was switched to freeze and the built-in S-Detect mode was initiated. From the US BI-RADS classification, the lesions were separated into six categories (0–5), with Category 4 further subdivided into 4A–C. Ultimately, for each US group (conventional, S-Detect, and Co-Detect), the lesion was diagnosed as either benign or malignant.

### Criteria of US image diagnoses

In the conventional US group, the two-dimensional gray-scale images were evaluated according to the BI-RADS lexicon, in which certain sonographic characteristics were suspicious for malignancy: irregular shape and edge, nonparallel expansion and growth, signal attenuation posteriorly, and microcalcifications. In the scoring system, lesions with one of the above characteristics were classified as 4a; lesions with two characteristics were classified as 4b, and lesions with ≥3 features were classified as 4c (10–12). Scores of ≤4a were considered benign and scores of ≥4b were considered malignant. The S-Detect group diagnoses were assigned according to the pre-programmed definitions: Category 3 (probably benign, 98%); Category 4 [suspicious, 2%–95%; Category 4A (low suspicion, >2%–10%), Category 4B (moderate suspicion, >10%–50%), and Category 4C (high suspicion, >50%–95%)]; and Category 5 (highly suggestive, >95%). For the Co-Detect group, the conventional B-mode scan and S-Detect method were synchronized and the final scores reflected modification of the 2D-US score according to whether the S-Direct was assigned to “Malignant” or “Benign” as illustrated in the table below. (For example, if the 2D-US score was 3 and the S-Detect assignment was “Malignant”, the final score was changed to 4a. If the S-Detect assignment was “Benign”, the score would remain a 3. Similarly, if the 2D-US score was 4b, but the S-Detect assignment was “Malignant”, the score would be upgraded to 4c, whereas if the S-Detect was “Benign”, the score would be downgraded to 4a.)

**Table:** 

2D-US BI-RADS score	S-Detect assignment	
	Malignant	Benign
3	4a	3
4a	4b	3
4b	4c	4a
4c	5	4b
5	5	4c

Results acquired as followed: Scores <4b (benign), scores ≥4b (malignant).

The gray-scale two-dimensional US images of each lesion were performed and re-evaluated by two independently experienced radiologists (with more than 10 years’ working experiences), under blinded conditions. An associate or chief physician was consulted if the diagnosis was not consistent, and a final co-diagnosis was achieved to minimize personal biases.

### Statistical analysis

Statistical analysis was performed through SPSS 22.0. Pathological diagnosis was regarded as the gold standard. MedCalc software was conducted to simulate the receiver operating characteristic (ROC) curve, and the area under curve (AUC) values of each curve was calculated. *Z*-test values were used to assess the differentiation of AUC values between groups (*p* < 0.05 means a significant difference).

## Results

### Pathological results

Of the 326 nodules, 87 were malignant (76 infiltrative ductal carcinoma, 6 intraductal carcinoma, 3 papillocarcinomas, and 1 Paget’s disease) and 239 were benign (106 fibroadenosis with fibroadenoma, 66 fibroadenosis, 38 fibroadenomas, 7 sclerosing adenosis, 11 intraductal papillomas, 5 granulomatous lobulitis, 5 fibrocystic diseases, and 1 adenomyoepithelioma) (**Table 1**).

**Table 1:** Pathological results and BI-RADS scores of lesions detected.

Pathological type	No.	BI-RADS scores					BI-RADS Post-adjustment (Co-Detect group)				
		3	4a	4b	4c	5	3	4a	4b	4c	5
**Benign**	239	70	153	14	2	0	200	22	10	5	2
Fibroadenosis with fibroadenoma	106	28	74	4	0	0	94	7	3	2	0
Fibroadenosis	66	19	43	4	0	0	53	8	5	0	0
Fibroadenoma	38	14	23	1	0	0	35	3	0	0	0
Sclerosing adenosis	11	3	5	2	1	0	6	1	1	2	1
Intraductal papilloma	7	2	5	0	0	0	6	1	0	0	0
Granulomatous lobulitis	5	0	3	2	0	0	2	1	1	1	0
Fibrocystic mammary disease	5	4	0	1	0	0	4	1	0	0	0
Adenomyoepithelial adenosis (adenomyoepithelioma)	1	0	0	0	1	0	0	0	0	0	1
**Malignant**	87	2	19	33	29	4	8	0	14	33	32
Infiltrative ductal carcinoma	76	0	16	28	28	4	5	0	12	28	31
Intraductal carcinoma *in situ*	6	1	1	4	0	0	1	0	1	4	0
Papillocarcinoma	3	1	0	1	1	0	0	0	1	1	1
Paget’s disease	1	0	1	0	0	0	1	0	0	0	0

### Diagnostic results of conventional US, S-detect, and co-detect groups

**Figure 1:**
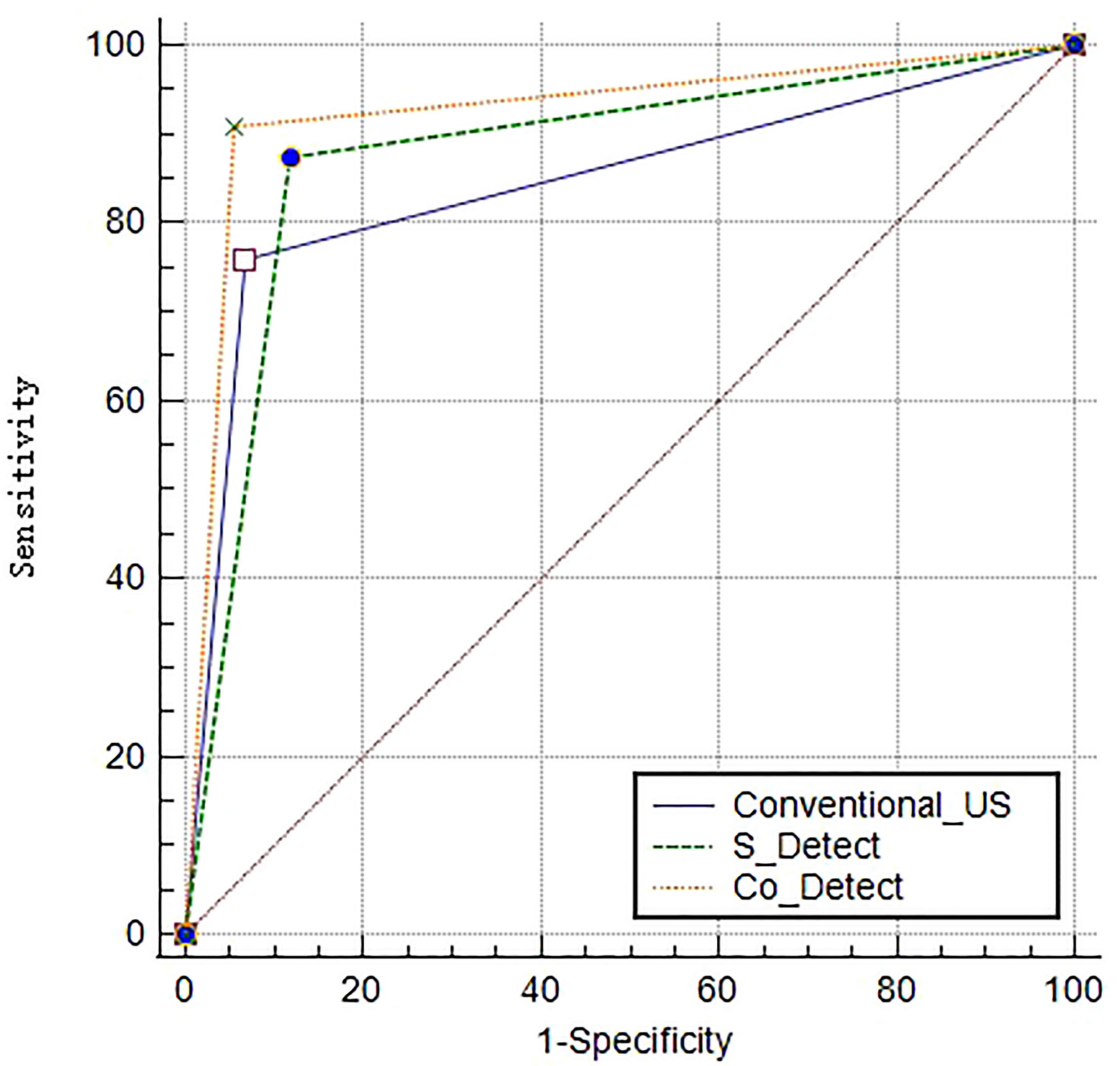
Receiver operating characteristic curve (ROC) and area under curve (AUC) values of the conventional US group, the S-Detect group, and the Co-Detect group.

**Figure 2:**
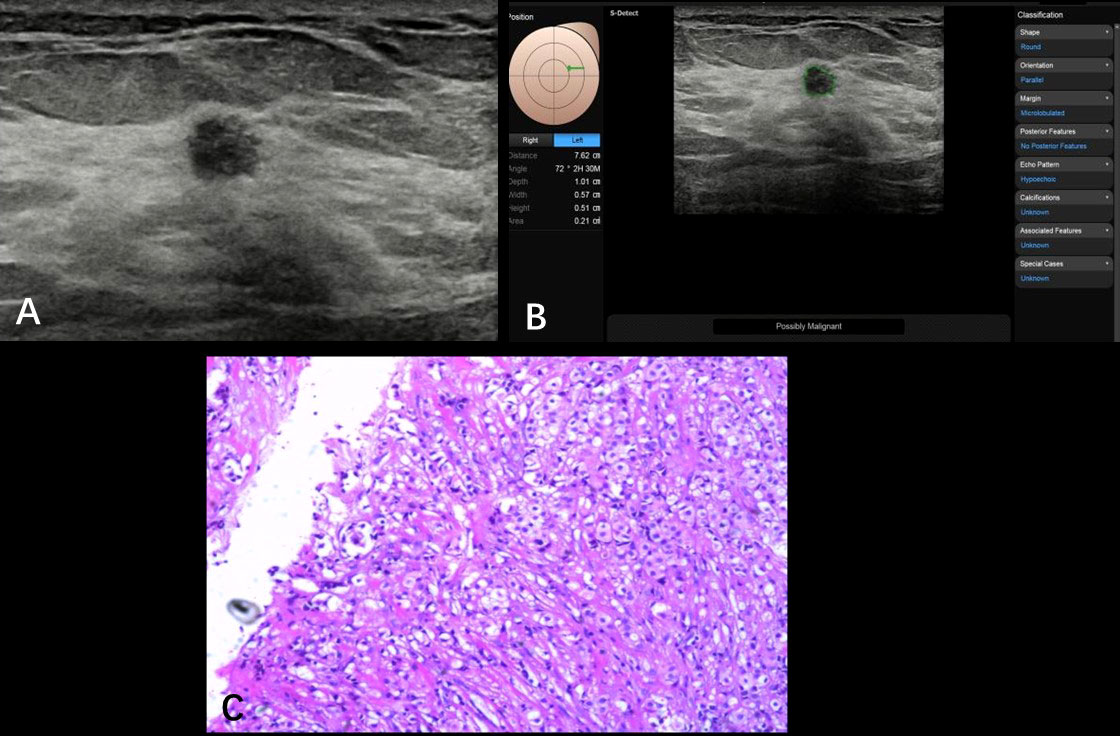
Non-specific invasive carcinoma from a 69-year-old patient (BI-RADS 4a). A hypoechoic nodule (0.57 × 0.51 cm) was found at the 2 o’clock point of her left breast; the ultrasound graphic showed an uneven edge, irregular shape, and uniform echo **(A)**. S-Detect artificial intelligence recommendation was “possibly malignant” and the BI-RADS score receded from 4a to 4b **(B)**. Pathologically confirmed as breast non-specific invasive carcinoma **(C)**.

**Figure 3:**
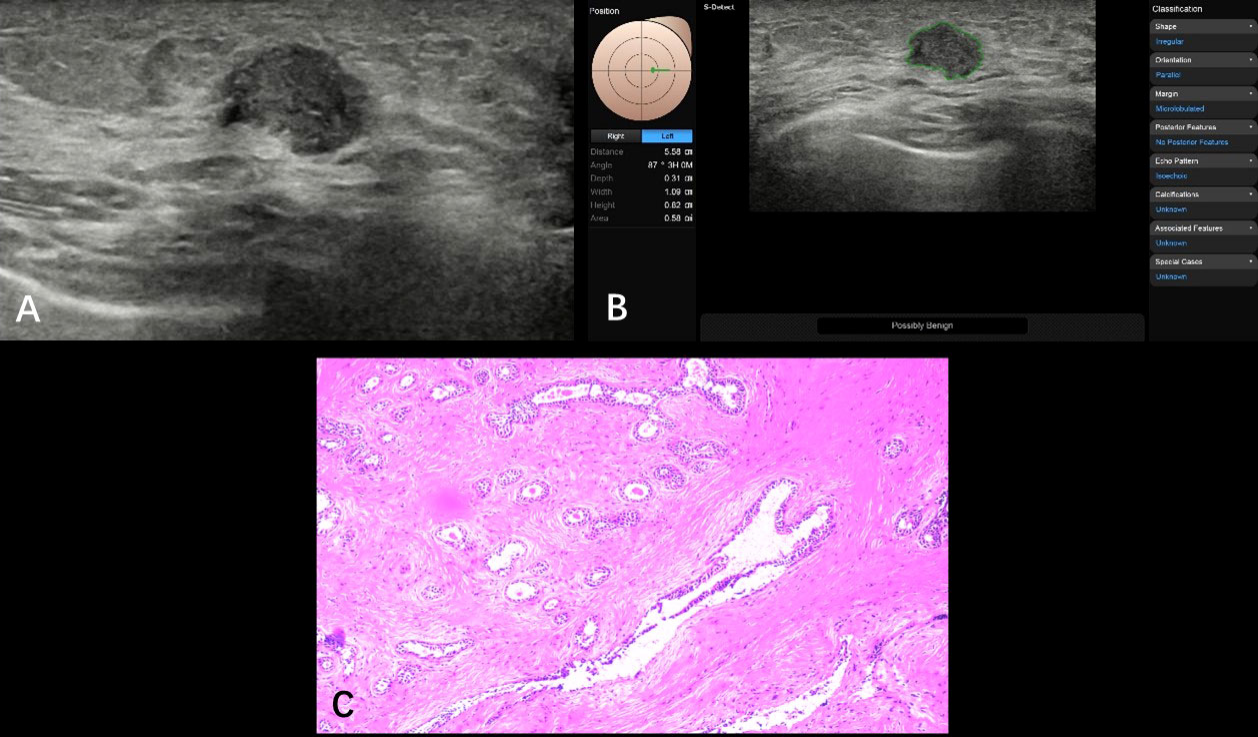
Fibroadenosis with fibroadenoma formation from a 35-year-old patient (BI-RADS 4a). A hypoechoic nodule (1.09 × 0.82 cm) was found at the 3 o’clock point of her left breast; ultrasound graphic showed glossy edge, irregular shape, partly angulated, and nonuniform internal echo **(A)**. S-Detect artificial intelligence recommendation was “possibly malignant” and the BI-RADS score receded from 4a to 4b **(B)**. Pathologically confirmed as breast fibroadenosis with fibroadenoma formation **(C)**.

**Figure 4:**
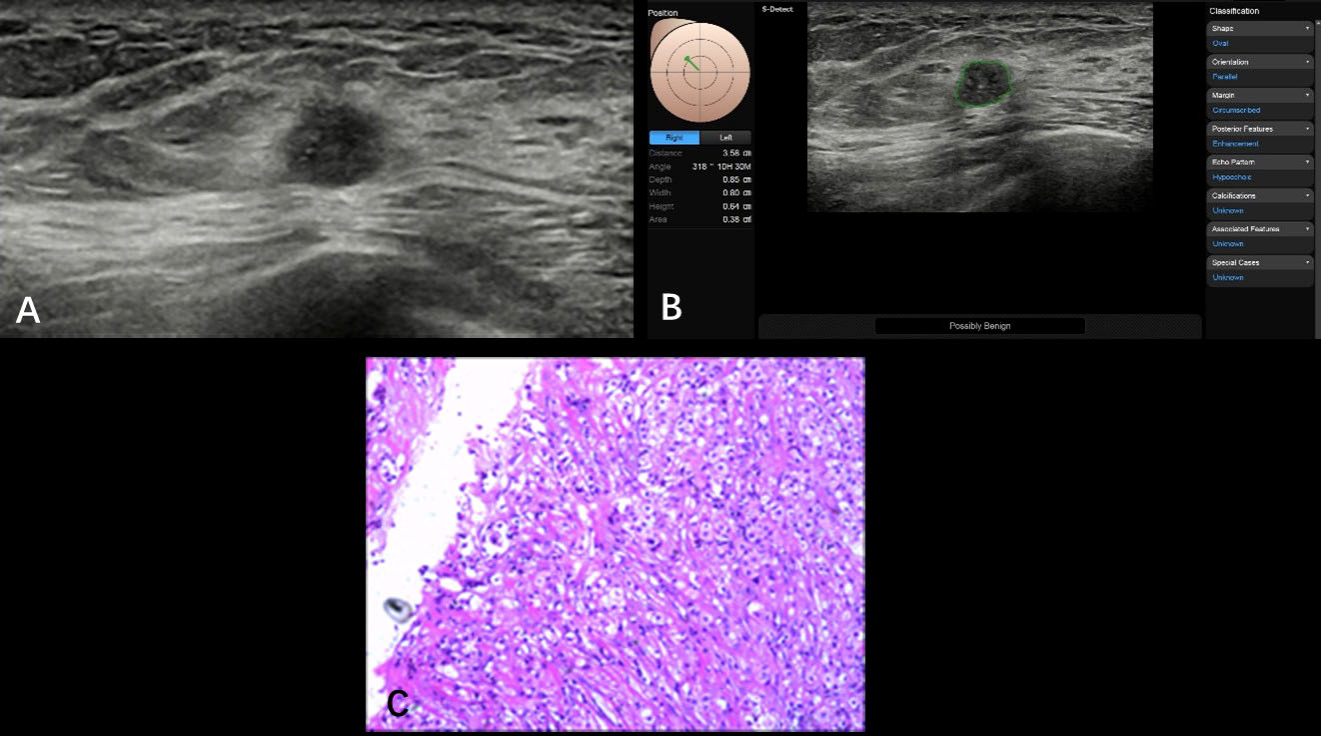
Non-specific invasive carcinoma from a 49-year-old patient (BI-RADS 4b). A hypoechoic nodule (0.86 × 0.68 cm) was found at the 10 o’clock point of her right breast with smooth and irregular edges; the inner echoes detected with nonuniform distribution came with spotted hyper-echoes **(A)**. The S-Detect suggestion was “possibly benign”; the BI-RADS classification was adjusted from 4b to 4a **(B)**. Pathological results are showed in **(C)** (HE, ×100). S-Detect gave a misdiagnosis due to the misrecognized microcalcifications that were found through operators.

**Figure 5:**
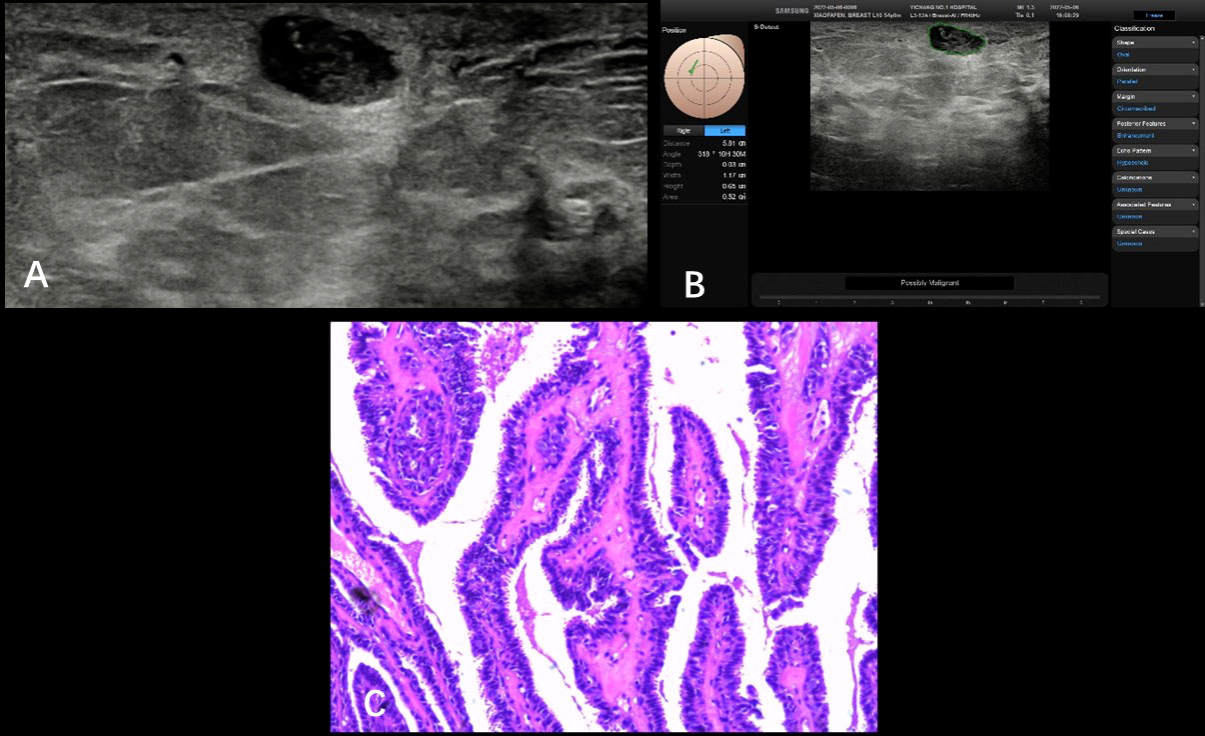
Intraductal papilloma from a 54-year-old patient (BI-RADS 4a). A hypoechoic nodule (1.12 × 0.65 cm) was detected at the 10 o’clock point of her right breast with smooth and irregular/regular edges (S-Detect technology failed to draw the outline of this nodule), nonuniform distribution with some spotted hyper-echoes **(A)**. The S-Detect suggestion was “possibly malignant” and BI-RADS score declined from 4a to 4b **(B)**. Pathology result was intraductal papilloma shown in the picture (HE, ×100) **(C)**. Inaccurate boundary layer distributed inappropriate malignant.

Diagnoses of the conventional US group included 244 benign and 82 malignant nodules with sensitivity, specificity, and accuracy values of 75.86%, 93.31%, and 88.65%, respectively (**Figure 1**). The S-Detect system assigned 222 benign and 104 malignant lesions with sensitivity, specificity, and accuracy values of 87.36%, 88.28%, and 88.04%, respectively. The Co-Detect group had diagnoses of 234 benign and 82 malignant nodules with improved sensitivity, specificity, and accuracy values of 90.80%, 94.56%, and 93.56%, respectively. Representative sonographic images and corresponding histology pictures are shown in **Figures 2**–**5**. Correlation of pathological results and BI-RADS scores initially and in the Co-Detect group is depicted in **Table 1**, and diagnostic results with statistical comparisons are shown in **Tables 2**, **3**.

**Table 2:** Ultrasonic diagnosis of different detected methods.

		Pathological results		
Method		Malignant	Benign	Total
		(*n*= 87)	(*n*= 239)	
Conventional US	Malignant	66	16	82
	Benign	21	223	244
S-Detect	Malignant	76	28	104
	Benign	11	211	222
Co-Detect	Malignant	79	13	92
	Benign	8	226	234

**Table 3:** Efficacy comparison of different methods in diagnosis.

Method	Sensitivity (%)	Specificity (%)	Accuracy (%)	Positive predictive value (%)	Negative predictive value (%)
Conventional US	75.86 (66/87)	93.31 (223/239)	88.65 (289/326)	80.49 (66/82)	91.39 (223/244)
S-Detect	87.36 (76/87)	88.28 (211/239)	88.04 (287/326)	73.08 (76/104)	98.05 (211/222)
Co-Detect	90.80 (79/87)	94.56 (226/239)	93.56 (305/326)	85.87 (79/92)	96.58 (226/234)

### ROCs and AUC results of different methods

The Co-Detect group had better diagnostic efficiency with a higher AUC value than the conventional and S-Detect groups (Co-Detect *vs*. conventional US, *Z* = 3.882, *p* = 0.0001; Co-Detect *vs*. S-Detect, *Z* = 3.861, *p* = 0.0001), whereas there was no significant difference between the conventional US and S-detect group regarding diagnostic efficiency (*Z* = 1.294, *p* = 0.1956); the AUC values of the conventional US, S-Detect, and Co-Detect groups were 0.85, 0.88 and 0.93, respectively (**Figure 1**).

## Discussion

Although ultrasonography is a commonly used technique to help differentiate benign and malignant breast nodules, not until BI-RADS was introduced by the American College of Radiology was there a standardized terminology to describe the characteristics and final assessment categories of breast lesions (13, 14). Nevertheless, the inevitable overlap of benign and suspicious characteristics together with inexperienced sonographers can still compromise the accuracy of US diagnostic accuracy (15). The S-Detect computer-assisted algorithm, based on AI and neural networks, “learning” on a large amount of training data, is a built-in program on US machines and, independent of human input, analyzes the morphological characteristics of the breast nodule according to the BI-RADS lexicon. Ultimately, it provides a dichotomous evaluation: “possibly benign” or “possibly malignant” (16, 17). In the present study, only small breast nodules (diameter ≤2 cm) were enrolled. According to the BI-RADS standard, the sensitivity, specificity, and accuracy of the diagnosis of malignant nodules were 75.86%, 93.31%, and 88.65%, respectively. Of the 87 malignant nodules, 19 were scored BI-RADS 4a: 10 nodules with a blurred edge and 9 nodules with an irregular shape; these lesions had only a single feature suspicious for malignancy (irregular shape and edges; non-parallel expansion and growth, signal attenuation posteriorly, and microcalcifications) and, therefore, scored as BI-RADS 4a. Of the 239 benign nodules, 14 had a BI-RADS 4b score and 2 had a BI-RADS 4c score; these BI-RADS classifications mismatched with pathological results. These misdiagnoses may be attributed to small size (diameter ≤1 cm, contour and inaccurate microcalcification programming observed), atypical echo features, and uncomprehensive morphological manifestations (irregular-shaped, parallel or unparallel orientation, micro-lobulated and irregular margins, and complex echogenicity in mass) that could be found in both benign and malignant lesions.

Several studies have confirmed that S-Detect technology improves the diagnostic accuracy of breast nodules (18, 19). However, there are very few studies focused on small breast nodules, especially with a maximum diameter limited to 2 cm. Briefly, in the present study, of 326 lesions in 295 patients, 87 were proven malignant and 239 were benign. The S-Detect group suggested 104 malignant and 222 benign nodules; 39 were misdiagnosed histologically. The sensitivity, specificity, and accuracy values of the S-Detect group were 87.36%, 88.28% and 88.04%, respectively. Of 11 malignancies misdiagnosed as benign, 5 were smaller than 1 cm with clear boundaries, and 1 nodule had inner microcalcification. Possible reasons for the misdiagnoses may be related to a size too small to automatically analyze, and the known limitation of the S-Detect technology is that it cannot analyze microcalcification within nodules (**Figures 4A, B**). Some microcalcification overlooked by S-Detect can easily be identified by senior radiologists, and the diagnosis was amended to reflect this finding. There were 28 nodules misdiagnosed as malignant: 10 with fibroadenosis and fibroadenoma, 9 with only fibroadenosis, 5 with intraductal papillomas, 2 cases of granulomatous lobular mastitis, 1 case of an adenomyoepithelioma, and 1 with sclerosing adenosis. In this study, we used a multipurpose ultrasonic probe (L2-12A, 5–13 MHz) to extend usage latitude. The possible reason for these inaccuracies may be related to the location and depth of masses; ultrasonic frequency is negatively correlated with detection range and imaging resolution (20, 21). In some cases, the increased divergence of interpretations of mammary gland images (5 cases were declared malignant in the S-Detect group) and shape irregularity (19 cases) was considered a higher risk in the S-Detect group (micro-lobulated, angular, and showing other suspicious marginal changes) that caused the S-Detect program to errantly define the borders of the nodule, potentially misrepresenting the edge of the nodules, which resulted in misdiagnosis as malignant (**Figure 5**).

In the Co-Detect group, S-Detect technology was used to adjust the BI-RADS scores assigned by conventional US. Markedly important, of 92 malignant and 234 benign nodules, 86.63% (149 nodules) of the 172 nodules with a BI-RADS 4a score by conventional US were downgraded to a BI-RADS 3 score due to the benign assignment by S-Detect. As a result, the clinical management of these patients changed from biopsy to clinical follow-up. Eventually, 95.97% (143 of 149) were confirmed as benign pathologically; groups of patients who had the same strategy of a corrected BI-RADS category may be able to avoid biopsy, thus avoiding unnecessary surgery and reducing costs. Although few in number, of concern were the 6 of 149 (4.03%) that were confirmed as malignant during follow up. Correctly downgraded from BI-RADS 4b to 4a were 9 of 77 (19.15%) nodules that were confirmed benign pathologically. Additionally, 38 nodules with a 4b score were upgraded to class 4c and subsequently confirmed as malignant. Clearly, although not perfect, both upgrade and downgrade score adjustments prompted by S-Detect improved the diagnostic accuracy of two-dimensional ultrasonography.

The Co-Detect group had impressive success in differentiating benign and malignant nodules with sensitivity, specificity, and accuracy values of 90.80%, 94.56%, and 93.56%, respectively. However, notably, there were 21 misdiagnoses: 8 pathologically malignant nodules were misdiagnosed as benign, including 5 infiltrating ductal carcinomas, 2 intraductal carcinomas, and 1 papillary carcinoma. The possible reasons for these “misses” might be attributed to an inappropriate BI-RADS 4a score in the traditional US group and unmatched BI-RADS 3 score in the Co-Detect group, optimistically regarded as advanced in the S-Detect group, with four of the nodules being quite small, <1 cm, edge sophisticated, irregular, with no circumscribed shape, irregular with angular margins, or lobulated. There were 13 benign nodules misdiagnosed as malignant, namely, 4 cases of fibroadenosis with fibroadenoma, 4 cases of intraductal papillomas, 2 cases of granulomatous mastitis, 2 cases of fibroadenoma, and 1 case of adenomyoepithelioma. Several of these had concerning characteristics based on the conventional US including serrated edge, shape irregularity, consistent echo, and parallel growth, which may cause incorrect BI-RADS 4a score in conventional US, incorrect classification in S-Detect, and incorrect diagnosis as BI-RADS 4b in the Co-Detect group (as malignant lesions). The AUC of the Co-Detect group relative to diagnostic efficiency was significantly higher than conventional US and S-Detect by statistical analysis (*Z* = 3.882, *p* = 0.0001; *Z* = 3.861, *p* = 0.0001).

Although we concluded that S-Detect clearly had definite clinical value for the diagnosis of small breast nodules, there were still some limitations. Specifically, S-Detect cannot identify microcalcifications within nodules, an important indicator suspicious for malignancy, whereas experienced radiologists could identify these ambiguities. S-Detect automatic nodule identification, particularly if small or if the edges are indistinct, might cause inappropriate classification; the addition of blood flow information, elastography, and other parameters could further enhance diagnostic characterization (22, 23). The present study demonstrated that S-Detect technology could provide accurate analysis, avoiding subjective physician bias. The combination of S-Detect and conventional ultrasonography could amplify the advantages of both, improving diagnostic efficiency, increasing diagnostic confidence, and significantly reducing the frequency of misdiagnosis. Furthermore, this feasible strategy for the evaluation of focal breast lesions we had conducted was applied to a single-center study; it should be further certified using a multi-center study, and the ability to reduce the margin of experience, which varies from resident doctors to top doctors, should be evaluated.

## Conclusions

In conclusion, there was an unequivocal improvement in diagnostic accuracy of small breast nodules with the incorporation of S-Detect; its use in conjunction with conventional ultrasonography can be confidently recommended.

## Data availability statement

The raw data supporting the conclusions of this article will be made available by the authors, without undue reservation.

## Ethics statement

Written informed consent was obtained from the individual(s) for the publication of any potentially identifiable images or data included in this article.

## Author contributions

(I) Conception and design: BX, XC, DW; (II) Administrative support: YW, Y-KL; (III) Provision of study materials or patients: BX, XC; (IV) Collection and assembly of data: BX, SL; (V) Data analysis and interpretation: BX, XC. All authors contributed to the article and approved the submitted version.
